# A short, animated storytelling video to reduce addiction stigma: A pilot randomized controlled trial

**DOI:** 10.1016/j.abrep.2025.100622

**Published:** 2025-06-17

**Authors:** Maxwell Klapow, Misha Seeff, Maya Adam, Merlin Greuel, Daniel Hoffman, Jessica R. Rogge, Andrew Gordon, Till Bärnighausen, Doron Amsalem

**Affiliations:** a Department of Experimental Psychology, University of Oxford, Oxford, UK; b United World College Maastricht, Maastricht, the Netherlands; c Dept. of Pediatrics, Stanford University School of Medicine, Stanford, CA, USA; d Heidelberg Institute of Global Health, Heidelberg University, Heidelberg, Germany; e Center for Digital Health, Dept. of Medicine, Stanford Medicine, Stanford, CA, USA; f Mathematical Institute, University of Oxford, Oxford, UK; g Boston University, College of Arts and Sciences, Boston, MA, USA; h Prolific Academic, London, UK; i Department of Global Health and Population, Harvard T. H Chan School of Public Health, Boston, USA; j Africa Health Research Institute (AHRI), Somkhele, KwaZulu-Natal, South Africa; k Dept. of Psychiatry, Columbia University Vagelos College of Physicians and Surgeons, NY, USA

**Keywords:** Addiction, Animated storytelling, Short, SAS, Stigma, Online trial, Video

## Abstract

**Background and aims:**

Public stigma towards people with addiction negatively impacts help-seeking, treatment and recovery. This pilot study tested the feasibility of conducting a large-scale, online trial to measure the effect of a short, animated storytelling (SAS) stigma reduction video, with and without soundtrack, on addiction stigma, optimism, warmth towards people with addiction, and hopefulness at two timepoints (immediately post-exposure and 14 days later).

**Design:**

We used a parallel group, three-arm randomized controlled trial (RCT).

**Setting:**

We conducted this fully online study on the Prolific Academic research platform (participant recruitment) and the Qualtrics survey platform (data collection).

**Participants:**

We recruited 631 English-speaking adult participants, aged 18–49, residing in the United States.

**Interventions:**

Intervention group A received the SAS video intervention. Intervention group B group received the SAS video intervention without sound. The control group received written information about global addiction prevalence, estimated to be time-equivalent with the video interventions.

**Measurements:**

We measured participant retention rate at the two-week follow-up to determine the feasibility of conducting the definitive trial. Our co-primary outcomes were addiction stigma, optimism, warmth towards people with addiction and hopefulness, measured using an abbreviated 18-item version of the Attribution Questionnaire (AQ-18), the Brief García’s Interactive Optimism Scale (BIOS-G), a stigma thermometer and a visual analogue scale (VAS). We used repeated-measures ANOVA to assess group-by-time interactions and compared changes from baseline to post-intervention. Participants completed follow-up surveys 14 days post-intervention.

**Findings:**

The retention rate from baseline to follow-up was 88.0 %. Exposure to both the video with sound and without sound resulted in significant positive changes compared to the control group, for pity [F (4,1046) = 3.26, η^2^ = 0.012, p = 0.011], willingness to help [F (4,1046) = 8.48, η^2^ = 0.031, p < 0.001], dangerousness [F (4,1046) = 2.95, η^2^ = 0.011, p = 0.019], avoidance [F (4,1046) = 4.25, η^2^ = 0.016, p = 0.002], as well as optimism [F (2,595) = 7.7, η^2^ = 0.014, p < 0.001], warmth toward people with addiction [F (2,594) = 6.5, η^2^ = 0.014, p = 0.002], and hopefulness [F (2,594) = 5.4, η^2^ = 0.013, p = 0.005]. No effects were observed for fear or blame stigma sub-scales. These effects were no longer visible at follow-up in this pilot sample. No significant differences were observed between the video with sound and the video without sound.

**Conclusions:**

This pilot study demonstrates the feasibility of proceeding with our registered, largescale, multi-country, online RCT. The significant effect observed in a relatively small pilot population, after a single exposure to this 2.5 min SAS intervention aimed at reducing addiction stigma, was unanticipated and is worthy of highlighting. A larger sample size will adequately power the full trial to detect both immediate effects and their potential durability over time, in various global settings.

## Highlights

• Fully online pilot RCT demonstrates feasibility for large-scale stigma reduction study.
• Single 2.5 min animated video significantly reduced addiction stigma post-view.
• High retention (88%) and minimal data issues indicate digital trial feasibility.
• Soundtrack and no-sound video arms showed similar immediate stigma reductions.
• SAS videos can be scalable for social media to reduce public addiction stigma.

## 1 Introduction

People with addiction and other substance use disorders face significant public stigma that negatively impacts help-seeking, treatment and recovery (Krendl & Perry, 2023). Addiction stigma fuels prejudice and discrimination towards affected individuals, as well as aggravating avoidance, self-stigmatization, and reluctance to seek care (P. W. Corrigan, 2017).This social phenomenon, which has been documented around the world and across cultures, exacerbates a growing public health challenge (Krendl & Perry, 2023). In 2019, the World Health Organization estimated that 400 million people over 15 years of age were struggling with alcohol use disorders (World Health Organization, 2024). Drug use disorders affect approximately 39.5 million people between the ages of 15 and 64 (World Health Organization, 2024), and their prevalence was aggravated globally by the COVID-19 pandemic (Kola et al., 2021, World Health Organization, 2021). The pandemic also exacerbated confounding problems like social isolation and loneliness, especially in under-served communities (World Health Organization, 2021). These challenges highlight the need for scalable, effective stigma-reduction interventions.

There are several challenges associated with stigma reduction in the public, largely related to the widespread perception of addiction as a moral failing rather than a treatable illness (P. W. Corrigan, 2017). Effective interventions need to shift prevailing public narratives around addiction (Barry et al., 2014). Misconceptions about addiction persist even among healthcare workers, often leading to substandard care for affected individuals (Van Boekel et al., 2013). These findings underscore the need for innovative approaches to the dissemination of hopeful and positive narratives related to addiction and substance abuse, thereby fostering empathy for affected individuals (Batson & Ahmad, 2009).

Social contact with people who are recovering from addiction has proven effective for reducing stigma towards them, although social contact-based interventions face logistical challenges that often prevent them from scaling broadly (Bielenberg et al., 2021). Individual selection bias often prevents social contact-based interventions from reaching the people who need them the most (Pettigrew, 1998). Language and cultural barriers further complicate the broad distribution of public health communication interventions (Piller et al., 2020). As a result, recent reviews examining the effectiveness of stigma-reduction interventions reported reductions in structural stigma in homogenous, high-income populations, but mixed findings beyond these settings (Livingston et al., 2012).

During the COVID-19 pandemic, a new approach to public health communication, called short, animated storytelling (SAS), was developed in order to spread important health information to diverse global audiences (Adam et al., Vandormael, 2021). Using relatable, animated characters, compelling soundtracks and a wordless, visual storytelling approach, SAS videos can overcome language, literacy and cultural barriers. Unlike traditional stigma-reduction interventions which often rely on didactic educational content or in-person contact strategies, SAS videos can deliver emotionally resonant narratives through universally understandable visual storytelling. This modality is especially advantageous in diverse, global contexts because it bypasses linguistic and literacy barriers, engages viewers through visual metaphors, and leverages familiar social media formats. This flexibility allows SAS content to be highly adaptable and thus more suitable for scale compared to traditional psychoeducational programmes and more wide-reaching than traditional public health messaging campaigns. Additionally, the brief, self-contained format of SAS videos allows for high dissemination potential and repeat exposure, which may be critical for maintaining stigma reduction effects over time.

Prior research suggests that these interventions can reach broad audiences, globally, via social media, where people increasingly seek health information in the post-pandemic era (Adam et al., n.d.; Cheng et al., 2021, Favaretti, 2022, Tsao, 2021, Vandormael, 2021). The rise in information-seeking via social media and the global reach of these platforms have transformed social media into an important avenue for the dissemination of public health messages (Tsao, 2021). However, if social media dissemination is to be harnessed to scale SAS interventions, these interventions need to first be rigorously tested, their efficacy proven, and their mechanisms of action explored.

Since SAS interventions are typically wordless, one potential mechanism of action could relate to the emotional engagement that is stimulated by their soundtracks. Research has suggested that music can promote positive sentiments towards a film’s story and boost the visual attention of its viewers (Millet et al., 2021). Examining such mechanisms of action is critical for supporting both engagement and the intended effects of SAS interventions.

This pilot study aims to determine the feasibility of conducting a large-scale, online experiment to measure the effect of a SAS stigma reduction video, with and without soundtrack, on addiction stigma, optimism, warmth towards people with addiction, and hopefulness at two timepoints (immediately post-intervention and 14 days later).

## 2 Methods

### 2.1 Setting, participants and eligibility

This study was conducted online. We recruited 631 participants living in the United States through the Prolific Academic research platform (ProA; https://www.prolific.co). We collected data from enrolled participants via the Stanford Medicine instance of Qualtrics, an online survey and data collection platform. Participants were English-speaking adults aged 18–49, with no additional restrictions placed on eligibility. This age group reflects our target audience as they are likely to seek information on social media. Broad eligibility criteria reflected our interest in piloting the intervention as it would be delivered outside of experimental conditions: online, widely available via social media.

### 2.2 Consent procedures

Before enrolling in the study, all participants read a study information sheet that had been approved by the Stanford University Internal Review Board (IRB). Participants who indicated their informed consent to participate were then directed to complete the study activities on Qualtrics, a secure online survey and data-collection platform.

### 2.3 Trial design

We conducted an individual, parallel group, three-arm randomized controlled trial. After consenting to take part in the study, participants were randomly assigned 1:1:1 into the three arms. Intervention A arm received the SAS video intervention. Intervention B arm received the SAS video intervention without any sound. The control arm received written information about global addiction prevalence, estimated to be time-equivalent with the video interventions (approximately 2.5 min).

**Fig. 1:**
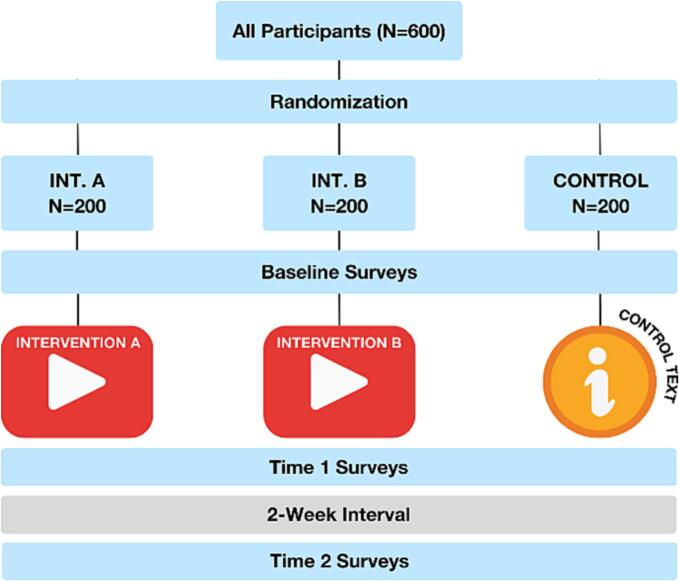
Trial design.

We collected demographic information and baseline data on our co-primary outcomes using a baseline survey. Immediately after exposure to the intervention or control condition, all groups completed a post-intervention survey assessing the co-primary outcomes. After 14-days, we administered a follow-up survey to assess the participant retention rate and explore the durability of any observed effects. We conducted attention checks to ensure validity of the data being collected and excluded participants who failed these checks. Each participant spent approximately 10 min on the intervention and surveys and received $4.80 for completing each part of the study. Fig. 1 shows the trial design.

### 2.4 Randomization and allocation concealment

We used a computer-generated allocation sequence to randomly assign participants in a 1:1:1 ratio to the three trial arms, using the Qualtrics participant randomization feature. The allocation sequence was concealed from the investigators, who remained unaware of individual trial arm allocations.

### 2.5 Intervention

**Fig. 2:**
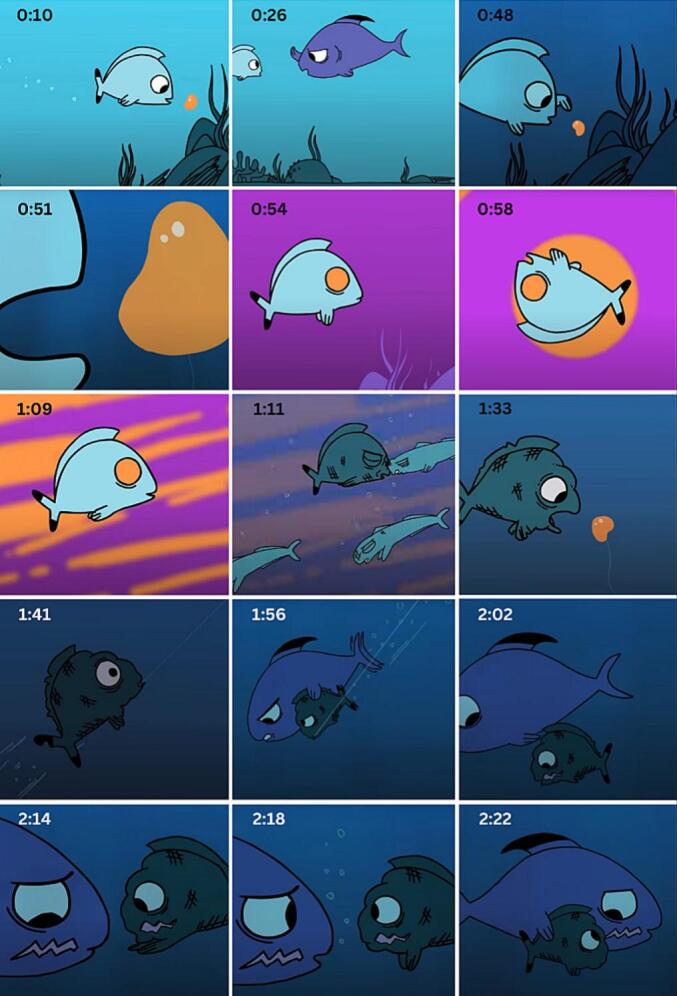
Selected scenes from SAS Intervention with timestamps.

The SAS video intervention was co-created with a global health communication specialist, an addiction medicine specialist, a professional animator, a composer, and an adolescent advisory group from the United States. The intervention portrays the addiction struggle of a young fish who discovers and tries a foreign substance, ignoring the warnings of an older fish. The young fish becomes addicted, and visual analogies depict the impact on his life. He harms other fish while under the influence and eventually hurts himself in reckless pursuit of the addictive substance as tolerance sets in. In desperation, he consumes the last of the substance before realizing he is hooked. A fishing line drags him off-screen, and it seems all is lost until the older fish reappears and helps him break free, although he is injured in the process. In the young fish’s moment of despair, the older fish reveals a similar injury, and the two share a moment of mutual empathy. The text on the final screen reads: “Hooked on something that’s hurting you? Speak up. There’s help.” Fig. 2 shows selected scenes from the SAS intervention video, with corresponding timestamps. The video can be viewed here: https://www.youtube.com/watch?v=a7LDlER67ZI.

#### 2.5.1 Sample size

The sample size target of 600 participants (200 per arm) was determined based on practical considerations for a pilot study primarily aimed at assessing the feasibility of a large-scale trial. This sample size was deemed sufficient to estimate retention rate at follow-up, ensure data quality and gather exploratory pilot data related to the effect of the intervention on our co-primary outcomes. Due to the exploratory nature of the pilot trial, we did not conduct formal power calculations (Cohen, 2013).

### 2.6 Primary outcome

#### 2.6.1 Feasibility

We measured participant retention rate at the two-week follow-up to determine the feasibility of a larger, definitive trial. Feasibility was the primary outcome of this pilot trial. We also examined data quality, as measured by the completeness of data collected and the proportion of participants who passed attention checks. Finally, we explored whether our primary measure of addiction stigma was sensitive to change given the short and light-touch nature of the intervention.

### 2.7 Co-primary outcomes

#### 2.7.1 Stigma towards people with addiction

Stigma was measured using an abbreviated 18-item version of the Attribution Questionnaire (AQ-18) (Brown, 2008). This version included six sub-scales, related to addiction stigma. These were:

1. Pity (the extent to which the respondent feels sorry for a person with addiction),
2. Willingness to help (the extent to which the respondent feels willing to help a person with addiction),
3. Dangerousness (the extent to which the respondent believes a person with addiction is dangerous),
4. Blame (the extent to which the respondent blames someone with addiction for their problem),
5. Avoidance (the extent to which the respondent feels inclined to avoid a person with addiction), and
6. Fear (the extent to which the respondent fears a person with addiction)

These sub-scales were selected based on the scope and content of the SAS intervention. The AQ-18 is scored along a 9-point Likert scale ranging from “not at all” (1) to “very much” (9), with a total maximum score of 27 for each 3-item construct, where greater scores indicate greater stigma. The questionnaire includes a brief vignette, which we modified to be gender-neutral:

“Alex is a 30-year-old with a history of addiction. Alex’s substance use problem has harmed Alex’s health and hurt others. Alex has been hospitalized several times for addiction-related health problems.”

#### 2.7.2 Optimism

We measured optimism using the Brief García’s Interactive Optimism Scale (BIOS-G), which assesses an individual’s level of general optimism. The scale includes 4 statements rated from 1 (“Of course not”) to 4 (“Yes, of course”), with higher scores indicating higher levels of optimism. The BIOS-G has demonstrated reliability and validity across diverse populations, with a reported Cronbach’s alpha of 0.86 (Garcia Cadena et al., 2021).

#### 2.7.3 Warmth towards people with addiction

Warmth toward people with addiction was assessed using a stigma visual analogue scale, a tool previously developed to measure attitudes toward stigmatized groups (Amsalem et al., 2022, Norton and Herek, 2013). Participants rated their personal feelings toward people with addiction on a scale from zero to 100, with higher scores indicating warmer or more favorable feelings. The visual analogue scale can be found in Supplementary Materials (S1).

#### 2.7.4 Hopefulness

We assessed participants’ subjective levels of hope using a visual analogue scale, which has been validated for assessing related constructs of stress and subjective well-being (Aitken, 1969, Lesage and Berjot, 2011). Participants rated how hopeful they felt after viewing the intervention, on a scale from zero to 100. The visual analogue scale can be found in Supplementary Materials (S2).

### 2.8 Statistical analysis

We performed data analysis using IBM SPSS Statistics 29. We used Pearson’s chi-square and one-way ANOVA to compare demographic characteristics (e.g., age, gender, ethnicity, race, political view, income, and education) between the three trial arms.

#### 2.8.1 Primary outcome: Feasibility

We assessed feasibility using descriptive statistics. We calculated retention rates as the percentage of participants who completed the intervention and follow-up assessments. To evaluate the quality of the data, we assessed the proportion of participants who passed attention checks and the completeness of all participants’ survey responses. We assessed sensitivity to change in measurement of stigma by comparing the changes in AQ-18 subscales between baseline and follow-up, across groups.

#### 2.8.2 Co-primary outcomes: Stigma towards people with addiction

We conducted a 3 × 3 repeated-measures ANOVA to compare the mean scores across sub-scales (pity, willingness to help, dangerousness, blame, avoidance, and fear, optimism, warmth, and hopefulness) among the three groups (video with sound, video without sound, and control) at three time points (baseline, post-intervention, and 14-day follow-up). Next, we used a 2 × 3 repeated-measures ANOVA to examine specific changes between baseline and post-intervention, as well as between baseline and 14-day follow-up, across the three groups. Finally, we conducted a one-way ANOVA to compare immediate changes among the three groups. We did not apply Bonferroni corrections due to the exploratory nature of this pilot study, as our primary aim was to identify potential trends and effects to inform future research rather than to control for multiple comparisons.

#### 2.8.3 Blinding

All co-investigators and researchers involved in data analysis remained unaware of the trial arm allocations throughout the study. Participants were unaware of the specific arm to which they were allocated.

### 2.9 Ethical considerations

This study was approved by the Stanford University Internal Review Board (IRB#76457) on 8/15/2024. We followed the CONSORT guidelines (Moher, 2010), and the study was registered on Open Science Framework (OSF) Registries (doi: 10.17605/OSF.IO/KX7Z8).

## 3 Results

### 3.1 Primary outcome

#### 3.1.1 Retention and participant flow

**Fig. 3:**
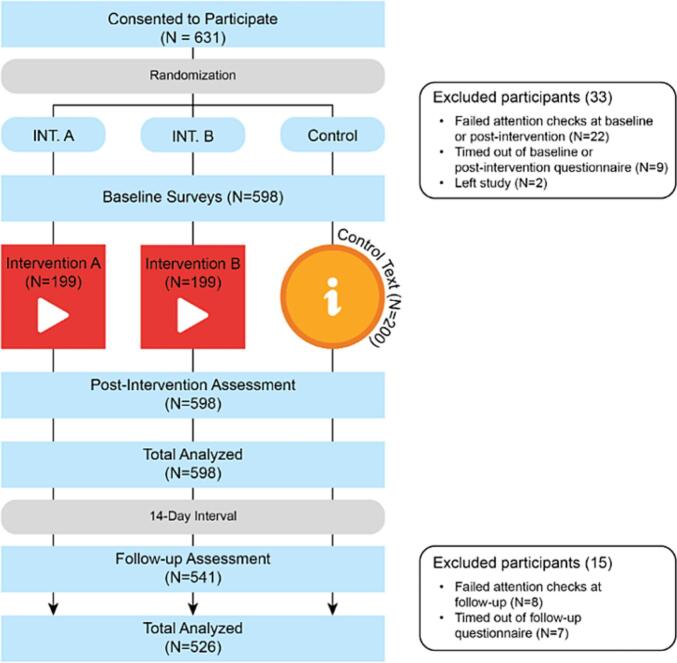
Participant flow diagram.

A total of 631 participants were recruited through the Prolific Academic online research platform and randomized into one of the three trial arms on Qualtrics. We excluded 22 participants (3.5 %) who failed attention tests, 9 participants who timed out of the first questionnaire (1.4 %) and 2 participants (0.0 %) who left the study. A total of 598 participants (94.8 %) successfully completed the baseline and immediate, post-intervention assessments. Of those, 526 (88.0 %) returned to complete the 14-day follow-up. Fig. 3 illustrates the participant flow diagram for the trial.

#### 3.1.2 Participant sociodemographic characteristics

Table 1 presents the sociodemographic characteristics. Completion rates were consistent across groups, and baseline characteristics were similar between follow-up completers and non-completers. The mean participant age was 34.1 ± 7.1 years (range 18–49). Approximately half of the participants identified as female (n = 289, 48.3 %) and male (n = 291, 48.7 %), with 18 participants (3 %) identifying as transgender, non-binary, or other. Regarding ethnicity, 10.2 % identified as Hispanic, and regarding race, 16.4 % identified as Black or African American, 68.2 % as White, 6.4 % as Asian or Pacific Islander, 0.7 % as Native American or Alaskan Native, and 11.8 % as mixed race or other. Within the sample, 42.0 % (251/598) of participants reported having personal experience with addiction (either past or present), 43.0 % (257/598) of participants reported significant childhood trauma, and 72.2 % (432/598) of participants indicated that a close friend or family member had struggled with addiction.

**Table 1:** Participant sociodemographic characteristics.

Demographics		Video with sound n = 200	Video without sound n = 202	Control n = 196	Statistic	
		*Mean (SD)*	*Mean (SD)*	*Mean (SD)*	*One-way ANOVA*	*p*
Age		33.78 (7.54)	34.82 (7.22)	33.57 (6.62)	1.92	0.147
Politics		40.85 (27.38)	41.23 (29.68)	35.7 (28.91)	2.13	0.120
		*n (%)*	*n (%)*	*n (%)*	*X^2^*	*p*
Gender					10.6	0.227
	Man	108 (54.0 %)	92 (45.5 %)	92 (46.9 %)		
	Woman	87 (43.5 %)	103 (51.0 %)	98 (50.0 %)		
	Transgender/Non-binary	3 (1.5 %)	7 (3.5 %)	5 (2.6 %)		
	Other	0 (0.0 %)	0 (0.0 %)	1 (0.5 %)		
	Prefer not to say	2 (1.0 %)	0 (0.0 %)	0 (0.0 %)		
	Total	200 (100.0 %)	202 (100.0 %)	196 (100.0 %)		
Hispanic/Latino					2.04	0.728
	Yes	24 (12.0 %)	19 (9.4 %)	18 (9.2 %)		
	No	175 (87.5 %)	183 (90.6 %)	177 (90.3 %)		
	Prefer not to say	1 (0.5 %)	0 (0.0 %)	1 (0.5 %)		
	Total	200 (100.0 %)	202 (100.0 %)	196 (100.0 %)		
Race						
	White / Caucasian	134 (67.0 %)	145 (71.8 %)	129 (65.8 %)	11.6	0.480
	Black or African American	32 (16.0 %)	34 (16.8 %)	32 (16.3 %)		
	Asian / Pacific Islander	9 (4.5 %)	10 (5.0 %)	19 (9.7 %)		
	Native American or Alaskan Native	2 (1.0 %)	1 (0.5 %)	1 (0.5 %)		
	Mixed Race	15 (7.5 %)	9 (4.5 %)	8 (4.1 %)		
	Other	4 (2.0 %)	1 (0.5 %)	4 (2.0 %)		
	Prefer not to answer	4 (2.0 %)	2 (1.0 %)	3 (1.5 %)		
	Total	200 (100.0 %)	202 (100.0 %)	196 (100.0 %)		
Income ($USD)					8.75	0.556
	$0-$30,000	43 (21.5 %)	47 (23.3 %)	38 (19.4 %)		
	$31,000-$60,000	51 (25.5 %)	45 (22.3 %)	64 (32.7 %)		
	$61,000-$90,000	52 (26.0 %)	51 (25.2 %)	40 (20.4 %)		
	$91,000-$120,000	20 (10.0 %)	27 (13.4 %)	24 (12.2 %)		
	$120,000+	27 (13.5 %)	28 (13.9 %)	24 (12.2 %)		
	Prefer not to say	7 (3.5 %)	4 (2.0 %)	6 (3.1 %)		
	Total	200 (100.0 %)	202 (100.0 %)	196 (100.0 %)		
Education					14.0	0.174
	Some high school or less	0 (0.0 %)	4 (2.0 %)	1 (0.5 %)		
	High school diploma or GED	36 (18.0 %)	30 (14.9 %)	24 (12.2 %)		
	Some college but no degree	35 (17.5 %)	30 (14.9 %)	49 (25.0 %)		
	Associates or technical degree	17 (8.5 %)	22 (10.9 %)	19 (9.7 %)		
	Bachelor's degree	75 (37.5 %)	76 (37.6 %)	70 (35.7 %)		
	Graduate or professional degree (MA, MS, MBA, PhD, MD, JD, DDS, etc.)	37 (18.5 %)	40 (19.8 %)	33 (16.8 %)		
	Total	200 (100.0 %)	202 (100.0 %)	196 (100.0 %)		
Religion					17.7	0.724
	Agnostic	42 (21.0 %)	34 (16.8 %)	41 (20.9 %)		
	Atheist	25 (12.5 %)	22 (10.9 %)	26 (13.3 %)		
	Buddhist	3 (1.5 %)	3 (1.5 %)	3 (1.5 %)		
	Hindu	0 (0.0 %)	0 (0.0 %)	2 (1.0 %)		
	Jewish	4 (2.0 %)	0 (0.0 %)	2 (1.0 %)		
	Mormon	2 (1.0 %)	2 (1.0 %)	3 (1.5 %)		
	Muslim	2 (1.0 %)	3 (1.5 %)	3 (1.5 %)		
	Orthodox such as Greek or Russian Orthodox	2 (1.0 %)	0 (0.0 %)	1 (0.5 %)		
	Protestant	38 (19.0 %)	43 (21.3 %)	37 (18.9 %)		
	Roman Catholic	31 (15.5 %)	38 (18.8 %)	22 (11.2 %)		
	Other	18 (9.0 %)	16 (7.9 %)	21 (10.7 %)		
	No religious affiliation	33 (16.5 %)	41 (20.3 %)	35 (17.9 %)		
	Total	200 (100.0 %)	202 (100.0 %)	196 (100.0 %)		

#### 3.1.3 Feasibility and acceptability

All participants who completed the baseline assessment viewed their assigned intervention or control condition without reporting any access issues. Specifically, participants in Intervention Group B (“without sound”) reported no concerns regarding the lack of sound, and participants in Group A (“with sound”) reported no problems hearing the soundtrack. No participants dropped out during or immediately following the intervention or control exposure, indicating adequate acceptability for all three conditions. During data collection, only 22 participants (3.5 %) failed attentions checks, an important indicator of data quality in online trials (Douglas et al., 2023). The BIOS-G showed good internal consistency for the present sample (α = 0.73). The follow-up retention rate was 88 %.

### 3.2 Co-primary outcomes

#### 3.2.1 Pity, willingness to help, dangerousness, blame, avoidance, and fear (AQ-18)

A repeated measures ANOVA revealed a significant group-by-time interaction for pity [F (4,1046) = 3.26, η^2^ = 0.012, p = 0.011], willingness to help [F (4,1046) = 8.48, η^2^ = 0.031, p < 0.001], dangerousness [F (4,1046) = 2.95, η^2^ = 0.011, p = 0.019], and avoidance scores [F (4,1046) = 4.25, η^2^ = 0.016, p = 0.002]. We observed no significant interactions for the blame or fear sub-scales in this pilot study. The results of our co-primary outcomes are presented in Table 2.

**Table 2:** Effectiveness of SAS intervention on stigma outcomes.

Study arm	Baseline		Post-intervention		Follow-up	*n*	*F*		Partial *η^2^*	*p*
	*Mean*	*SD*	*Mean*	*SD*	*Mean*	*SD*				
		Pity								
Video with sound	19.91	5.34	19.95	5.43	19.52	5.30	171	3.26	0.012	0.011*
Video without sound	20.66	4.71	20.71	4.89	19.90	5.51	178			
Control	20.01	5.40	19.19	5.76	19.41	5.42	177			
		Willingness to Help								
Video with sound	17.50	6.55	17.91	6.71	16.68	6.56	171	8.48	0.031	<0.001***
Video without sound	18.38	6.29	18.62	6.54	17.04	6.56	178			
Control	17.63	6.29	16.31	6.75	15.57	6.23	177			
		Dangerousness								
Video with sound	15.36	4.41	14.10	4.75	14.79	4.50	171	2.95	0.011	0.019*
Video without sound	14.88	4.05	13.83	4.34	14.56	4.51	178			
Control	15.76	4.30	15.55	4.55	15.74	4.29	177			
		Blame								
Video with sound	15.34	5.01	14.82	5.41	15.89	4.90	171	0.01	0.000	0.136
Video without sound	15.43	5.17	14.70	5.52	15.40	4.82	178			
Control	14.64	5.57	14.64	5.71	15.27	5.22	177			
		Avoidance								
Video with sound	14.12	5.94	13.20	5.79	13.88	5.89	171	4.25	0.016	0.002**
Video without sound	13.53	5.67	12.43	5.67	13.58	6.00	178			
Control	14.24	6.09	14.45	6.21	14.76	5.65	177			
		Fear								
Video with sound	13.62	5.83	12.87	5.98	12.60	5.66	171	2.18	0.001	0.069
Video without sound	12.85	5.53	12.10	5.95	12.57	5.77	178			
Control	13.31	6.18	13.47	6.52	13.47	6.17	177			

Method: Repeated-measures analysis of variance (ANOVA).

*p < 0.05. **p < 0.01. ***p < 0.001.

To better understand the outcome differences between *time points*, we compared changes from baseline to post-intervention assessment and from baseline to the 14-day follow-up. We observed a significant, immediate group-by-time effect for pity [F (2,595) = 7.0, η^2^ = 0.023, p = 0.001], willingness to help [F (2,595) = 15.9, η^2^ = 0.051, p < 0.001], dangerousness [F (2,595) = 6.8, η^2^ = 0.022, p = 0.001], and avoidance sub-scores [F (2,595) = 8.1, η^2^ = 0.027, p < 0.001]. At the 14-day follow-up, a significant lasting group-by-time effect was observed for the willingness to help sub-scale only [F (2,523) = 3.6, η^2^ = 0.014, p = 0.028]. Although some evidence of durability remained, the observed effect was no longer significant for the pity, dangerousness, or avoidance sub-scales at the 14-day follow-up.

**Fig. 4:**
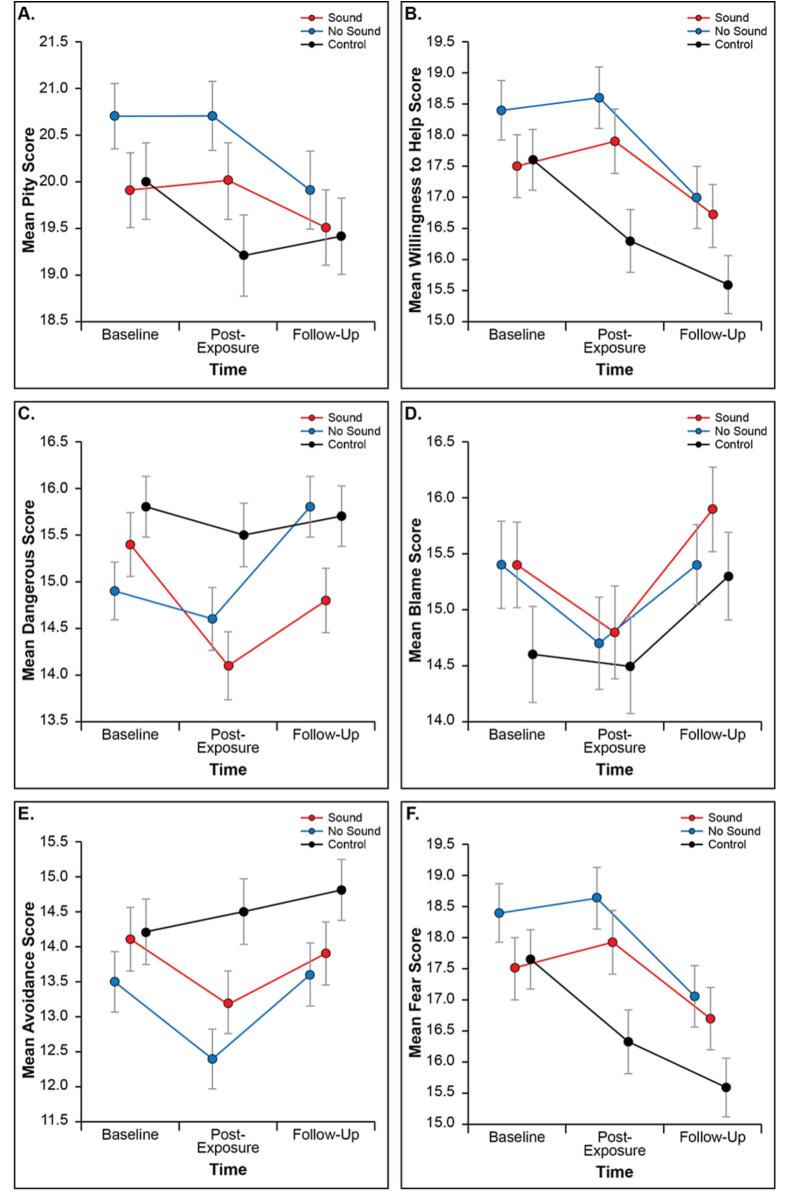
Stigma sub-scale group differences.

To better understand *group* differences, we compared immediate changes in outcomes. Both the video with sound and the video without sound outperformed the control group for pity (mean change: 0.80 [CI: 0.11–1.49], p = 0.017; 1.02 [0.41–1.63], p < 0.001, respectively), willingness to help (1.57, [0.82–2.32], p < 0.001; 1.47, [0.79–2.15], p < 0.001), dangerousness (1.08, [0.34–1.82], p = 0.002; 0.82, [0.18–1.47], p = 0.008), and avoidance scores (1.03, [0.21–1.84], p = 0.009; 1.27, [0.58–1.96], p < 0.001). No significant differences were observed, in this pilot study, between the video with sound and the video without sound. Fig. 4 shows the differences between groups for each of the six stigma sub-scales.

### 3.3 Secondary outcomes

#### 3.3.1 Optimism, warmth, and hopefulness

A repeated measures ANOVA revealed a significant group-by-time interaction for optimism [F (4,1050) = 3.8, η^2^ = 0.014, p = 0.004], warmth [F (4,1044) = 3.6, η^2^ = 0.14, p = 0.006], and hopefulness [F (4,1044) = 3.4, η^2^ = 0.013, p = 0.009]. Table 3 shows the group-by-time interactions for optimism, warmth towards people with addiction and hopefulness. To further explore differences across *time points*, we compared changes from baseline to post-intervention and baseline to the 14-day follow-up. Significant immediate group-by-time effects were observed for optimism [F (2,595) = 7.7, η^2^ = 0.025, p < 0.001], warmth [F (2,594) = 6.5, η^2^ = 0.021, p = 0.002], and hopefulness [F (2,594) = 5.4, η^2^ = 0.018, p = 0.005]. These effects did not remain significant at the 14-day follow-up, in this pilot population.

**Table 3:** Effect of the SAS interventions on optimism, warmth towards people with addiction, and hopefulness.

Study arm	Pre-intervention		Post-intervention		Follow-up		*n*	*F*	Partial *η^2^*	*p*
	*Mean*	*SD*	*Mean*	*SD*	*Mean*	*SD*				
		Optimism								
Video with sound	11.59	1.58	11.67	1.58	11.51	1.49	172	3.84	0.014	0.004**
Video without sound	11.58	1.39	11.72	1.32	11.56	1.53	178			
Control	11.68	1.35	11.46	1.41	11.38	1.28	178			
		Warmth Visual Analogue Scale								
Video with sound	53.71	21.86	57.78	22.31	54.02	23.68	170	3.65	0.014	0.006**
Video without sound	56.19	21.65	60.54	21.87	56.71	23.05	178			
Control	50.52	21.31	51.37	21.62	52.11	22.45	177			
		Hopefulness Visual Analogue Scale								
Video with sound	60.55	23.38	62.06	24.60	62.34	22.12	170	3.38	0.013	0.009**
Video without sound	62.93	21.92	65.84	22.17	66.10	21.05	178			
Control	62.83	23.01	61.06	22.43	64.66	20.15	177			

Method: Repeated-measures analysis of variance (ANOVA).

*p < 0.05. **p < 0.01. ***p < 0.001.

**Fig. 5:**
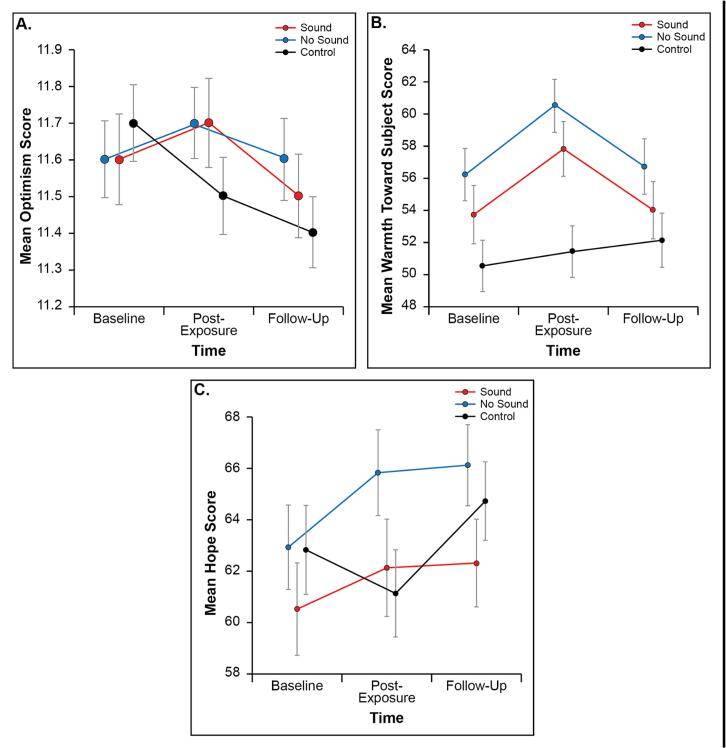
Group differences for optimism, warmth toward people with addiction, and hopefulness.

To better understand *group* differences, we compared immediate changes in outcomes. Both the video with sound and the video without sound outperformed the control group for optimism (0.30 [0.07–0.52], p = 0.005; 0.34 [0.13–0.55], p < 0.001, respectively) and warmth towards people with addiction (3.05 [0.45–5.64], p = 0.017; 3.67 [1.24–6.10], p = 0.001). For hopefulness, the video without sound outperformed the control group (3.98 [1.14–6.81], p = 0.003), while the video with sound showed a near-significant effect (2.72 [-0.02–5.47], p = 0.053). No significant differences were found between the video with sound and the video without sound in this pilot study. Fig. 5 shows the differences between groups for optimism, warmth toward people with addiction and hopefulness.

## 4 Discussion

The findings of this fully online pilot study demonstrate the feasibility of conducting a large-scale online trial to measure the effect of a short, animated storytelling intervention on addiction stigma in the public. An unanticipated finding, worth highlighting, is the fact that even in a relatively small pilot population, single exposure to the short (2.5 min) SAS video intervention significantly reduced addiction stigma immediately after exposure. To our knowledge, no other digitally scalable, social contact-based interventions have demonstrated such an effect for reducing addiction stigma. While the effect was no longer visible after 14 days, in this sample, the intervention’s replicable nature gives it the potential to be presented repeatedly, or itegrated into a series of similar videos that could feasibly reduce addiction stigma in the longterm.

Because of the short duration of SAS video interventions (∼2min), important questions remain about the durability of their effect after a single exposure. The larger sample size in the definitive trial will allow us a) to confirm the preliminary (immediate) effects we observed in the pilot trial, but also b) to determine whether the effect of this single SAS video remained durable after 14 days. A recent study examining the effect of a single-exposure SAS intervention on transphobia reduction also showed a significant immediate effect that had diminished 30 days after exposure (Amsalem et al., n.d.). Independent of the outcome of the definitive addiction stigma trial, these findings suggest the need to explore the effect of SAS “dosing”, either through multiple exposures to a single video or through testing of a *series* of SAS videos aimed at reducing addiction stigma.

Follow-up retention rates were high (88 %), especially given that this was a digital health intervention, many of which suffer from poor retention (Eysenbach, 2005). The high participant retention rates underscore the feasibility of conducting a definitive trial. Prior research suggests that low retention rates are one of the major stumbling blocks of digital health interventions. The high retention rate observed here could have been motivated by the fact that participants were paid for each study phase separately, only after completing each phase. Another possible explanation is that a large proportion of the participants had personal experience with addiction (42 %) or had a close friend or family member with addiction (72.2 %). These lived experiences could have motivated participants to return for the follow-up and, if true, this raises interesting questions about how we can optimize participants’ level of personal investment in online trials. Another indicator of feasibility was the low prevalence of participants (3.5 %) who failed attention checks during data collection. Attention checks have been identified as an important indicator of data quality in online trials (Douglas et al., 2023), and we received clear guidance from co-author AG on how to word these attention checks and how many to deploy. Other researchers have published attention check “best practices” (Gummer et al., 2021) that have been shown to dramatically improve the quality of data collected via online surveys.

While the main purpose of this pilot study was to establish the feasibility of the definitive trial, we also aimed to test the acceptability for participants of receiving an intervention that had no sound. We saw no evidence of confusion or early drop-off for participants enrolled in the no-sound arm, but we also saw very little difference between the preliminary observed effects in the full intervention arm vs. the no-sound arm. Both intervention arms appeared similarly effective, although there were some indications that the soundtrack may have enhanced the durability of the intervention effect. While the simple interpretation of this preliminary finding is that soundtracks may not matter that much, another interpretation was suggested by our co-author MG. He wondered whether many (or even most) of the participants assigned to the *full* intervention arm had actually watched the SAS video *without* the sound. In a world where people regularly consume short video content on their phones – possibly in public or noisy places – it is entirely possible that many participants in the “with sound” arm actually did not hear the soundtrack. This led us to decide to add specific written instructions, for the Intervention A arm in the large-scale trial, instructing participants to “turn on your volume now” as participants may be unaware that there is a sound component to their assigned intervention.

The observed effects on the stigma sub-scales in this pilot study also raise interesting questions about the differential effects of the intervention on different stigma sub-scales. These could be answered by a definitive trial. For example, perceived dangerousness and avoidance are central elements of addiction stigma (Rundle et al., 2021). The SAS video narrative highlights the lack of agency in the main character who becomes addicted. It is possible that the intervention narrative may have shifted viewers’ beliefs that addiction is inherently a moral failing, thereby eliciting a larger effect on these specific components of stigma (P. Corrigan et al., 2003). In addition to the planned large-scale trial, future research in this area might involve a performance evaluation that would allow us to explore the mechanism of action of SAS videos aimed at reducing addiction stigma.

Prior research has consistently identified public stigma as a major barrier to addiction treatment and recovery (Wakeman & Rich, 2018). Although many interventions have been tested, most focus on educational approaches (Bland et al., 2001), and contact-based initiatives, which can be costly and difficult to scale (Livingston et al., 2012). The current intervention is designed for wide dissemination and is suitable for implementation on social media platforms or other mass-media modes of delivery. Our findings demonstrate the potential of SAS interventions to be used as a first-line approach for population-level reduction of addiction stigma.

### 4.1 Limitations

Two limitations of this pilot study should be noted. First, as the study primarily focused on feasibility, it was not adequately powered to definitively measure the effects of the intervention nor conduct subgroup analyses. Second, participants were recruited on the Prolific Academic platform within the US, which limits the generalizability of the present findings. Cultural differences and variation in how stigma is conceptualized may influence the effect of the intervention in different parts of the world. This is one of the key motivations for conducting a multi-country trial as a next step.

### 4.2 Implications and future research

The preliminary findings from this pilot trial carry several important implications for the development and implementation of scalable stigma-reduction interventions. First, the immediate reductions in addiction stigma following a single, brief exposure to an SAS video suggest that emotionally engaging, narrative-based content can effect change even in short formats. While the effects were not sustained at the 14-day follow-up, the high feasibility and acceptability of the intervention support the potential for repeat exposures or sequential video series to sustain impact. Second, the negligible difference in outcomes between the full intervention and the no-sound version indicates the need for understanding user context (e.g. checking volume settings) and confirming implementation fidelity when conducting a larger, definitive trial.

Future research should explore optimal “dosing” strategies and delivery mechanisms for SAS content, including personalization and cultural adaptation across regions. Additionally, a large, high-powered trial will allow for a closer study of mechanisms of action to understand effectiveness and guide future SAS-based interventions. Further study of SAS interventions and their underlying mechanisms can help inform a broader agenda for integrating scalable, digital tools into global public health efforts to reduce addiction stigma.

### 4.3 Conclusion

The findings of this fully online, pilot study support the feasibility of proceeding to conduct a large-scale, registered, multi-country RCT to test the effect of an SAS video intervention on addiction stigma in adults living in different global regions. The study also provides unanticipated preliminary evidence that single exposure to the ∼ 2.5 min SAS video intervention can immediately reduce addiction stigma, while boosting optimism, warmth towards people with addiction and hopefulness. The larger sample size recruited for the definitive study will adequately power the trial: a) to confirm the preliminary effects we observed in this pilot study, b) to measure any potential lasting effects of this SAS intervention after 14 days, and c) to quantify the contribution of a soundtrack to both the effect of the intervention and the durability of that effect. Additionally, the definitive trial will provide more granular data on how this particular SAS intervention may differentially effect various stigma sub-scales. Conducting the definitive trial across three global regions will also allow us to study the differential effects and the global scalability of SAS interventions aimed at reducing addiction stigma. As the prevalence of addiction increases globally, there is an urgent need to develop and test accessible and globally scalable interventions, like SAS, to reduce addiction stigma and promote help-seeking.

*Registration:* The study was registered on Open Science Framework (OSF) Registries (doi: 10.17605/OSF.IO/KX7Z8).

## CRediT authorship contribution statement

**Maxwell Klapow:** Writing – original draft, Writing – review & editing, Methodology, Project administration, Investigation, Data curation, Formal analysis, Conceptualization. **Misha Seeff:** Writing – original draft, Writing – review & editing, Software, Investigation, Conceptualization. **Maya Adam:** Writing – original draft, Writing – review & editing, Visualization, Supervision, Methodology, Investigation, Conceptualization, Funding acquisition. **Merlin Greuel:** Writing – review & editing, Methodology. **Daniel Hoffman:** Writing – review & editing, Data curation, Formal analysis. **Jessica R. Rogge:** Writing – review & editing, Visualization, Investigation, Conceptualization. **Andrew Gordon:** Writing – review & editing, Software, Investigation, Methodology, Conceptualization, Data curation. **Till Bärnighausen:** Supervision. **Doron Amsalem:** Writing – original draft, Writing – review & editing, Visualization, Methodology, Investigation, Data curation, Formal analysis.

## Funding

Funding for this pilot randomized controlled trial was provided by the Stanford Center for Digital Health.

## Declaration of competing interest

The authors declare that they have no known competing financial interests or personal relationships that could have appeared to influence the work reported in this paper.

## Data Availability

The data that support the findings of this study are available from the corresponding author upon reasonable request.
