# Supplementary figures and images for: A short, animated storytelling video to reduce addiction stigma: A pilot randomized controlled trial
Source: Addict Behav Rep. 2025 Jun 17;22:100622. doi: 10.1016/j.abrep.2025.100622 (PMC12214250; doi:10.1016/j.abrep.2025.100622)

Supplementary File 1

**Stigma Visual Analogue Scale**

**
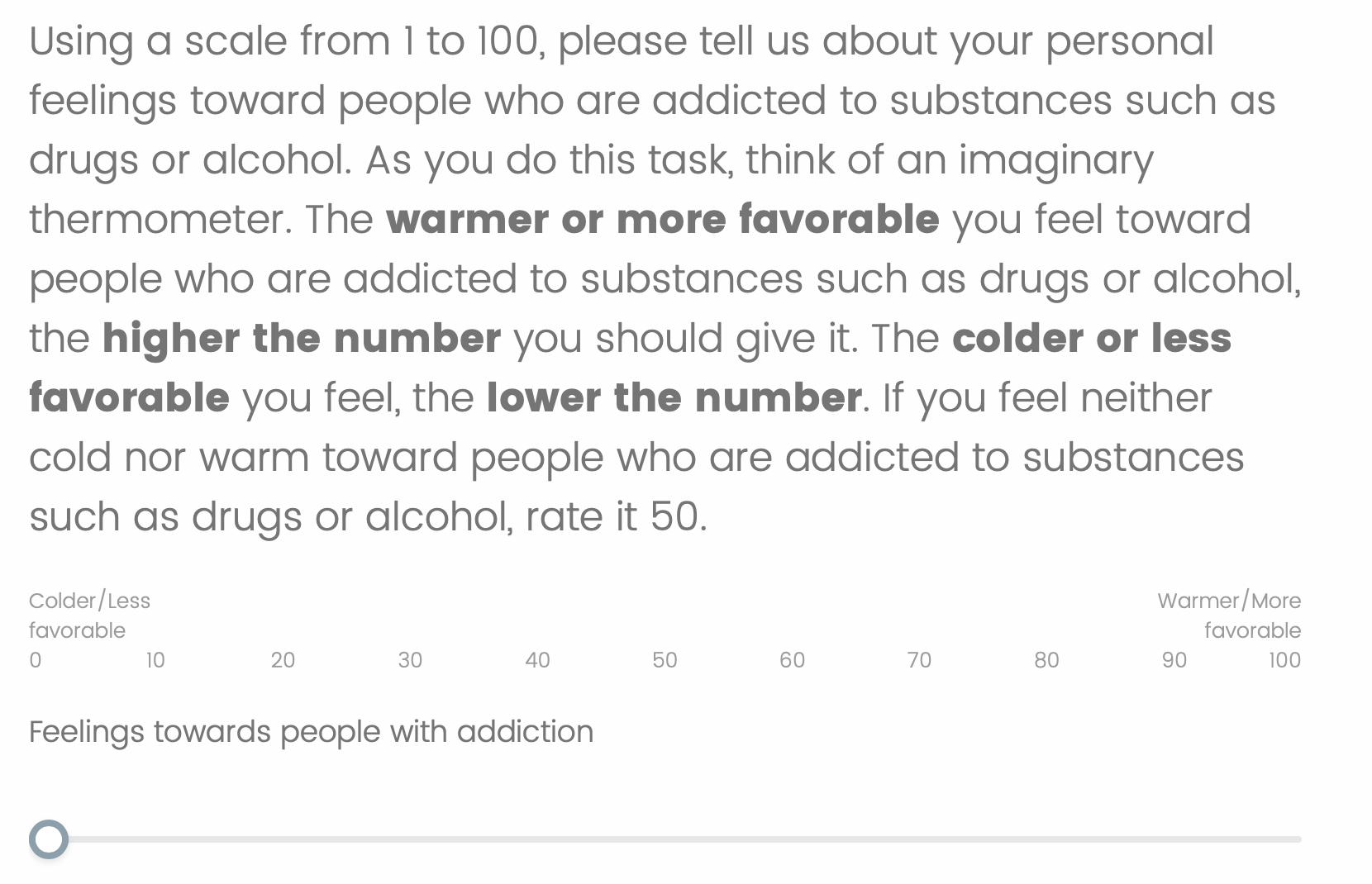
**

Supplement: Supplementary Data 1 [file mmc1.docx]

Supplementary File 2

**Hope Visual Analogue Scale**


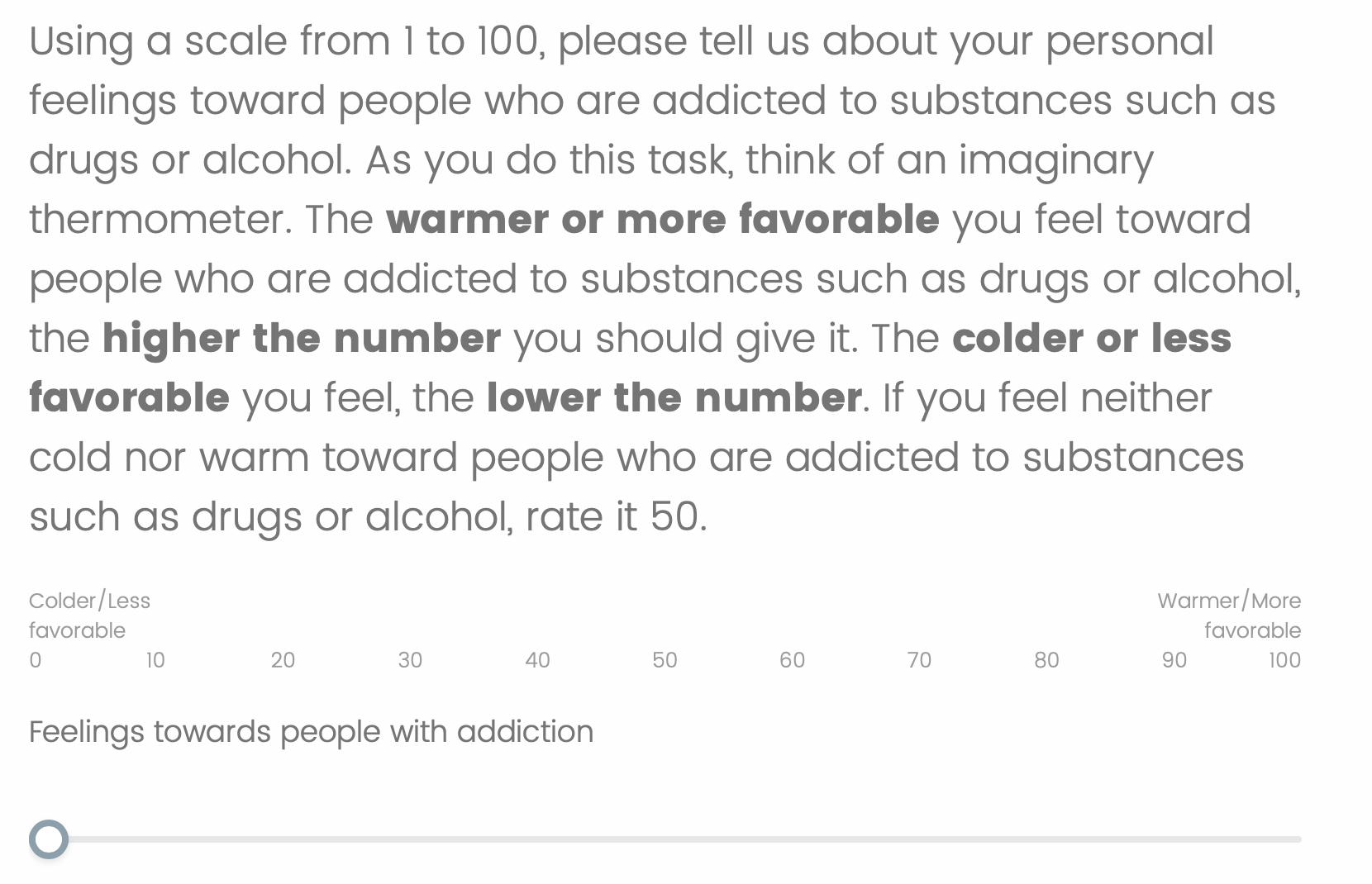

Supplement: Supplementary Data 2 [file mmc2.docx]
